# Prevalence of adverse childhood experiences and their co-occurrence in a large population of adolescents: a Young HUNT 3 study

**DOI:** 10.1007/s00127-022-02277-z

**Published:** 2022-04-22

**Authors:** Rosalie Broekhof, Hans M. Nordahl, Sigrid Bjørnelv, Sara G. Selvik

**Affiliations:** 1 Department of Psychiatry, Namsos Hospital, Nord-Trøndelag Hospital Trust, Namsos, Norway; 2 Department of Mental Health, Faculty of Medicine and Health Sciences, NTNU-Norwegian University of Science and Technology, Trondheim, Norway; 3 St. Olavs University Hospital, Avd. Østmarka, Trondheim, Norway

**Keywords:** ACE, Childhood maltreatment, Abuse, Neglect, Prevalence

## Abstract

**Purpose:**

Studies of adverse childhood experiences (ACEs) undertaken at the time of adolescence in the general population are not common. The aim of this study was to determine the prevalence and co-occurrence of the individual ACEs and sub-types of ACEs in a large population of adolescents.

**Methods:**

Data were used from the Young Nord-Trøndelag Health (Young HUNT 3) study, a population-based study of young adolescents. ACEs were operational defined as sexual, physical and/or emotional abuse; physical and/or emotional neglect; and/or household dysfunction. Co-occurrence was measured as the accumulation of ACEs and as an overlap analysis.

**Results:**

Of the 8199 evaluable adolescents, 65.8% had experienced at least one ACE and 28% of those had experienced more than one ACE. Household dysfunction was the most prevalent ACE subtype. The biggest overlaps among the three ACE sub-types were seen in those reporting neglect or abuse.

**Conclusion:**

There was a high degree of overlap between the three ACE sub-types and the individual ACEs, indicating that ACEs should be assessed together as a whole rather than separately. This study provides an opportunity to assess ACEs and their co-occurrences in relation to outcomes later in life.

## Background

The impact and severity of adverse childhood experiences (ACEs) were largely underestimated until research from the 1990s showed that ACEs had severe short- and long-term consequences for mental and physical health [1–4]. In addition to the association of ACEs with health issues, such as cardiovascular diseases and premature death [1, 4], there is also an association with mental disorders, such as mood, anxiety, schizophrenia, impulse control and substance use disorders [5–9]. Patients with histories of ACEs seek care more often, make high use of healthcare services, and have poor quality of life [10]. In a report from 2016, the World Health Organization (WHO) stated that it is a challenging task to study the scope of childhood maltreatment [11] because the prevalence of childhood maltreatment varies for different countries and numbers are often underestimated [11]. Variations in reported numbers might be explained by variations in the type of ACE studied, variations in the characteristics of the sample studied, or variations in the definition of ACEs. For example, the prevalence of reported child sexual abuse varied from 7 to 36% for women and 3 to 29% for men in one report [12]. However, the WHO concluded that at least 12% of children were sexually abused in 2015 [13]. The same report stated that 25% of all adults reported physical abuse in childhood [13]. In 2009, Gilbert et al. reported a prevalence of 4 to 16% for physical abuse and around 10% for neglect of children in high-income countries [14].

The WHO defines childhood maltreatment as “the abuse and neglect of people under 18 years of age” [11]. “It includes all forms of physical and/or emotional ill-treatment, sexual abuse, neglect or negligent treatment or commercial or other exploitation, resulting in actual or potential harm to the child’s health, survival, development or dignity in the context of a relationship of responsibility, trust or power” [11]. The adverse childhood experiences (ACEs) study by Felitti and Anda were the first study that defined ACEs in more detail [1, 15]. ACE scores were assessed using (combined) questionnaires that followed the WHO’s definition of childhood maltreatment [11], and household dysfunction was added as another form of maltreatment. Later studies on ACEs used assessment methods from the original ACE study [16], which assessed five types of interpersonal violence/childhood maltreatment: (a) sexual abuse; (b) physical abuse; (c) emotional abuse (also referred to as psychological abuse); (d) neglect and (e) household dysfunction [1]. Neglect can be divided into (d-1) physical neglect and (d-2) emotional neglect (see definitions in Table 1).

**Table 1:** Definitions of adverse childhood experience (ACE) types

Type of ACE	Definition
Sexual abuse	Defined by the occurrence of sexual touching or fondling, attempted intercourse, or actual intercourse by any adult or other person when the child did not want the act to occur or was too young to understand what was happening
Physical abuse	Defined by the occurrence of a parent or other adult who pushed, grabbed, shoved, slapped, or hit the child; or hit the child so hard it left marks or bruises, or caused an injury
Emotional abuse	Defined by the occurrence of a parent or other adult who swore at, insulted, or said hurtful things to the child; or threatened to hit or throw something at the child (but did not do it); or acted in any other way that made the child afraid he/she would be physically hurt or injured
Physical neglect	Defined as the child being left unsupervised when too young to care for themselves or going without needed clothing, school supplies, food, or medical treatment
Emotional neglect	Defined as the child not feeling part of a close-knit family or there being no one in the child’s family of origin who made the child feel special, wanted the child to succeed, believed in the child, or provided strength and support
Household dysfunction	Defined by the presence of life stressors, such as parental divorce, drug use by parents, psychiatric problems of parents, financial problems

The ACE study found that 52% of those assessed had experienced at least one individual ACE and 27% had experienced two or more ACEs [1]. This shows the complexity and interactive character of ACE. More recent research has focused on the specific areas of the different individual ACEs. For example, sexually abused girls had more learning problems, more symptoms of depression and anxiety and lower self-esteem than those who had not been sexually abused [17]. In another study, women with a history of sexual abuse had a higher risk of a very broad spectrum of psychopathology, such as major depression, anxiety, and alcohol and drug dependence [18]. Physical abuse in childhood has been associated with increased frequency of depression, anxiety, anger, physical symptoms and a number of medical diagnoses, decades after the abuse [19]. Emotional neglect has been related to depression, dysthymia and social phobia, with the likelihood of developing more than one lifetime affective disorder [20]. Neglect is the least studied type of trauma [14].

Again, other studies focused only on the sum of the individual ACEs [21–23]. It was found that individuals with a high sum of individual ACEs were at increased risk for sexual risk taking behavior, mental ill health, and problematic alcohol use [21–23]. This risk was even stronger for problematic drug use and interpersonal and self-directed violence [21–23].

Previous studies on ACEs have limitations. First, they tend to focus mainly on only one individual ACE [17–19]. Without measuring a broad range of ACEs, any long-term influence might be wrongly attributed to an individual type of abuse, neglect or household dysfunction. Second, the questionnaires were often based on retrospective reporting [1, 3, 15, 24]. Retrospective reports are vulnerable to recall bias [25]. While the ACE study did focus on the whole range of ACEs, the study population had presented for preventive health evaluations and were given questionnaires relating to retrospectively remembered ACE [1, 15, 26]. Thirdly, the early literature shows a dose–response relationship between an increase in the number of ACEs and poorer adult mental health [3, 15, 21, 24]. With only the number of ACEs, the ACEs interaction are not exposed.

## Aims

This study is part of a more comprehensive research project. The overall aim of the project is to assess ACEs in association with adult mental health. The aims of this study are:

- to assess the prevalence of ACEs and their co-occurrence measured at the time of adolescence in a large population sample. ACEs will be operationally defined in the Young Nord-Trøndelag Health 3 questionnaire.
- to add to the collective understanding of the prevalence and co-occurrence of ACEs, measured as the accumulation of ACEs and an overlap analysis of the three sub-types of ACE at the time of adolescence.

## Methods

### Study population

The county of Nord-Trøndelag in Norway has 135,000 inhabitants. All the inhabitants were invited to join a large population study: the HUNT study. The purpose of the HUNT study was to gather information on a wide variety of conditions and lifestyle factors. Data and samples were obtained in three waves of data gathering: 1984–1986, 1995–1997 and 2006–2008. A new wave of data gathering has recently been started.

The epidemiological study of adolescents in the region was named Young HUNT. The third wave of this study (the Young HUNT 3 study) was conducted between 2006 and 2008; 10,464 adolescents were invited to participate [27].

In the Young HUNT 3 study, during a school hour, the students completed a self-administered questionnaire that contained over 100 health- and lifestyle-related questions, including items on exposure to traumatic events, loneliness, psychological distress, and family cohesion. In total, 8199 adolescents (78%) filled in the questionnaires: 4129 girls (50.4%) and 4070 (49.6%) boys. The mean age was 15.91 (SD 0.03) years for the girls and 15.85 (SD 0.03) years for the boys.

The study was approved by the Norwegian Research Committee for Medical and Health Research Ethics (REK 2016/1877). Inclusion was based on written consent from participants aged 16 years and older and from parents for those under 16 years, in accordance with Norwegian law.

### Definition of adverse childhood experiences (ACEs)

**Fig. 1:**
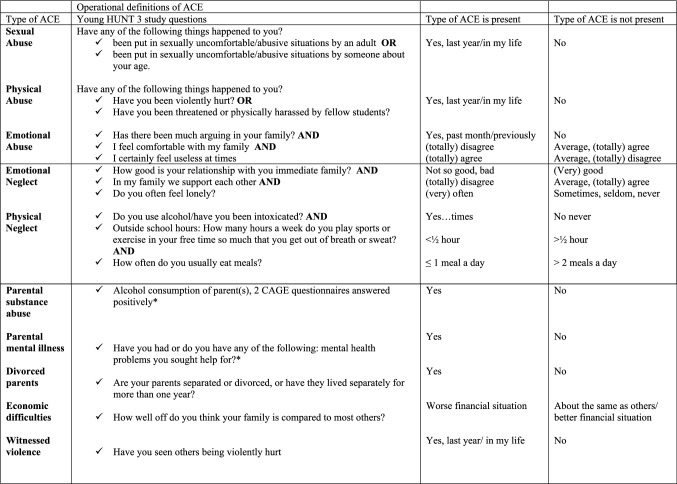
Overview of the questions from the Young Helse Nord-Trøndelag (HUNT) study questionnaire to obtain operational definitions of adverse childhood experiences (ACEs) * Questions for parents of the Young HUNT 3 study adolescents who had participated in the HUNT 3 study

The operationalization of ACEs was done by consensus of a group of experts in the HUNT material. These experts followed the outlines given in the ACE study by Felitti et al. [1], using the corresponding items in the questionnaire in the Young HUNT 3 study. To form an operational definition of household dysfunction, Young HUNT 3 participants were linked to their parents, who had participated in the HUNT 3 study, through a national family register code from Statistics Norway (SSB). See Fig. 1.

### Co-occurrence of the ACEs

Co-occurrence was measured in two different ways. First, as the accumulation of individual ACEs and the accumulation of ACEs within a subtype. Second, as the overlap between the three sub-types of ACE, visualized using a Venn diagram.

### Statistics

The ACEs were operationalized by combining the dichotomized variables, in a similar fashion to that of Fellitti et al. [1]. The prevalence of the individual operational ACEs and the total scores for abuse, neglect and household dysfunction were calculated to account for potential cumulative effects. We used SPSS version 23.0 software for the descriptive analyses.

## Results

In total, 88% of the adolescents lived together with their parents and 68% had siblings. The distribution of living in rural or urban situations was 72% versus 28%.

### Prevalence of ACEs

**Fig. 2:**
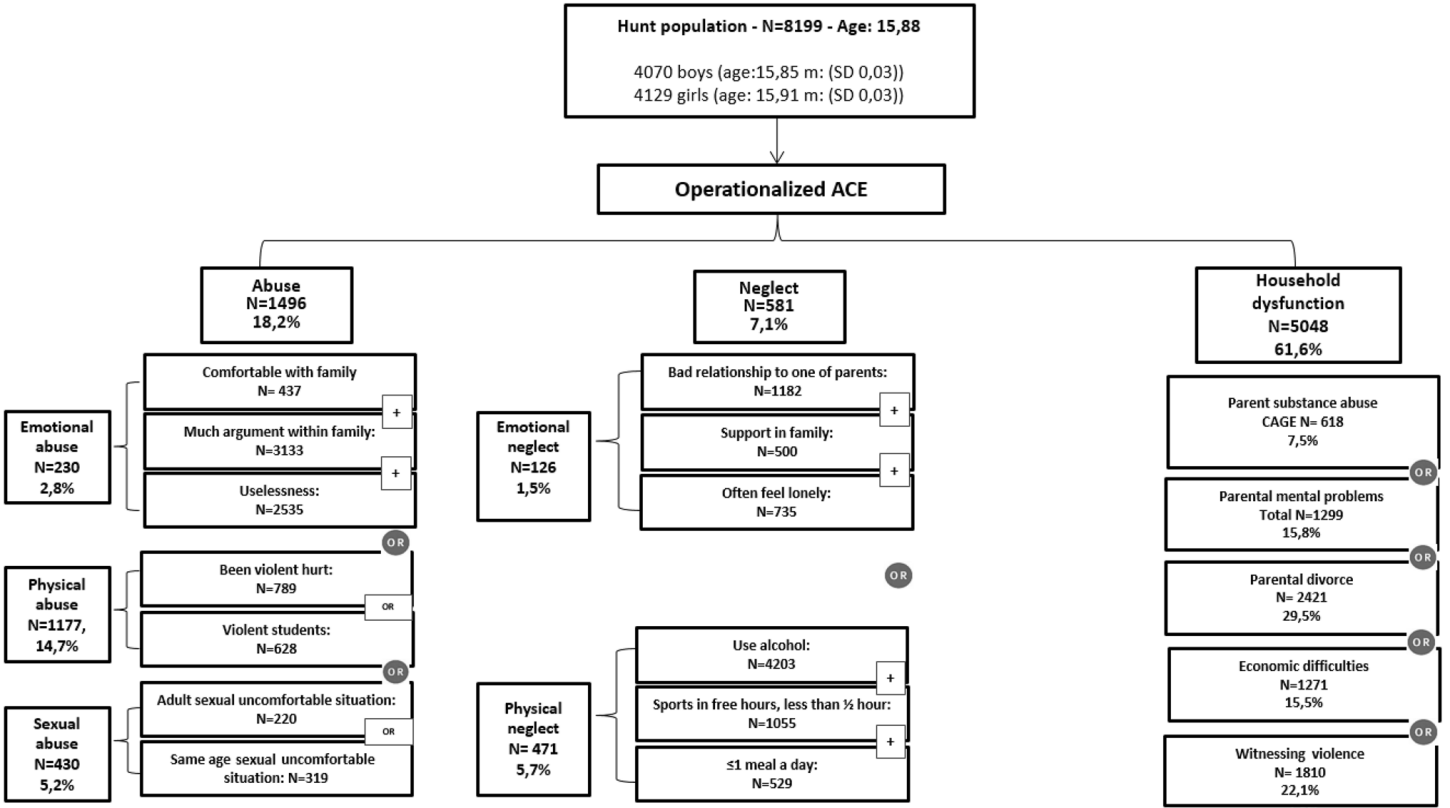
Operational definitions of adverse childhood experiences (ACEs). The prevalence is given as the number of adolescents responding positively (*N*) and also as a percentage of the total number of evaluable adolescents in the Young HUNT 3 study (*N* = 8199; 10,464 were invited) The significant higher ACEs for girls are emotional and sexual abuse, emotional neglect, parental substance use, and parental divorce. The significant higher ACEs for boys are physical abuse and witnessing violence. *m *mean, *SD *standard deviation, *y* years. * Questions for parents of the Young HUNT 3 study adolescents, who had participated in the HUNT 3 study

Figure 2 gives an overview of the operational definitions of the ACEs.

Of the entire sample (*N* = 8199), household dysfunction had the highest percentage of positive responses (*N* = 5048, 61.6%). Of household dysfunction, parental divorce was most prevalent (*N* = 2421, 29.5% of the entire sample), followed by having witnessed violence (*N* = 1810, 22.1%), parental mental problems (*N* = 1299, 18.8%), economic difficulties (*N* = 1271, 15.5%), and parental substance abuse (*N* = 618, 7.5%). More parental substance abuse and parental divorce were seen for girls and more witnessed violence for boys. Abuse in some form was reported by 1496 (18.2%) adolescents. Of abuse, physical abuse was most prevalent (*N* = 1177, 14.7%), followed by sexual abuse (*N* = 430, 5.2%) and emotional abuse (*N* = 230, 2.8%). Girls reported significantly higher levels of emotional and sexual abuse and boys, significantly higher physical abuse. Neglect was reported by 581 (7.1%) adolescents: 126 reported emotional neglect and 471 reported physical neglect. Girls reported significantly more emotional neglect than boys.

### Co-occurrence of the ACEs

**Fig.3:**
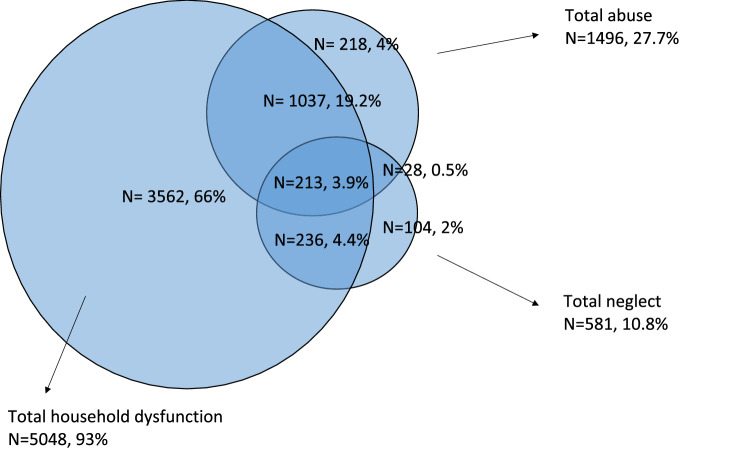
Venn diagram showing overlap between the three sub-types of adverse childhood experience (ACE). Results are given as the number of adolescents responding positively and as percentages of the number of adolescents in the Young HUNT 3 study who had experienced at least one of the three sub-types of ACE, *N* = 5398

The Venn diagram in Fig. 3 shows the overlap of reports of the three sub-types of ACE. Of the 8199 adolescents who completed the questionnaire, 65.8% had experienced at least one subtype of ACE and 28% of these had experienced two or more sub-types of ACE (Fig. 3). Of those who had experienced at least one subtype of ACE (*N* = 5398), 4% had experienced all three sub-types. The biggest overlaps between the sub-types of ACE were seen for neglect and abuse. For example, of the adolescents who experienced neglect, 104 experienced only neglect, while 477 also experienced another of the subtype of ACEs.

Table 2 shows the accumulation sores for the sub-types of ACEs as well as the accumulation scores for the individual ACEs. Of those who had experienced abuse (*N* = 1496), 283 (19%) had experienced two sub-types and 29 (1.9%) had experienced all three. Sixteen (2.8%) adolescents experienced more than one subtype of neglect.

**Table 2:** Overview of the accumulation sores for the sub-types of ACEs and the accumulation scores for the individual ACEs

(Sub)types	1	2	3	4	5	≥ 6
Abuse *N* = 1496	1184 (79.1%)	283 (19.0%)	29 (1.9%)			
Neglect *N* = 581	565 (97.2%)	16 (2.8%)				
Household dysfunction *N* = 5048	3219 (63.8%)	1350 (26.7%)	420 (8.3%)	55 (1.1%)	4 (0.1%)	
Accumulation of individual ACEs SE = 1,8 *N* = 5398	2771 (51.3%)	1460 (27.0%)	705 (13.1%)	314 (5.8%)	103 (1.9%)	49 (0.9%)

Percentages were calculated from the total number of adolescents (*N*) who had experienced that type of ACE

For household dysfunction, 3219 (63.8%) adolescents had experienced one subtype, 1350 (26.7%) had experienced two sub-types, and 479 (9.5%) had experienced three or more sub-types.

The average accumulation score for ACE, calculated from the total of all individual ACEs, was 1.8. Of those who had experienced ACE (*N* = 5398), around 50% of the adolescents had experienced at least one type of individual ACE, almost 30% two, and 20% three or more individual ACEs.

## Discussion

Our results show that in a non-clinical population study of adolescents aged 13–19 years, the prevalence of ACEs was high, and the ACEs often occurred together. In our sample, 65.8% of the participants (*N* = 5398) had experienced at least one subtype of ACE, and 28% (1514) of those had experienced more than one subtype. Household dysfunction was the most prevalent subtype of ACE. Abuse and neglect had the biggest overlaps with the other sub-types of ACEs.

This high prevalence of ACEs is in line with earlier retrospective studies in adults, which found that over 50% had been exposed to at least one individual ACE and around 30% had been exposed to two or more individual ACEs [1, 3, 24]. A study by McLaughlin et al. also assessed 13- to 17-year-olds [7]. They defined childhood adversities as parental loss, maltreatment, parental maladjustment and economic adversity, and found that 58.3% of the participants had experienced at least one individual childhood adversity and 59.7% of these had experienced multiple types.

The exposure rates for abuse and neglect in our study are in the lower range of those in earlier studies of adults [1, 14, 15, 28, 29]. As mentioned, most of those studies of ACEs were carried out retrospectively [1, 3, 15, 24, 26] and included other notions/reflections on the questions than those for the children aged 13–19 years in our study.

The questionnaire in our study sourced data that covered all but two of the household dysfunction events defined in the ACE study [1]. The adolescents in the Young HUNT study were not asked if their mothers were treated violently or about incarcerated household members. The Young HUNT questionnaire did enquire whether the adolescent had ‘witnessed violence’, including outside the family. We also expanded the household dysfunction individual ACEs by adding parental divorce and economic difficulties. Subsequently, we found a higher prevalence rate for household dysfunction in general than found in the ACE study [1] but in line with McLaughlin’s study [7] and the expanded ACE study [15]. In contrast, the prevalence of witnessed violence in Philadelphia, retrospectively assessed in the same way as in our study, was twice as high [29].

That ACEs interact with other ACEs is not a new concept [1, 21]. The “ACE score”, the sum of ACEs, has received comments on its simplicity and research is encouraged to focus also on the concept of timing, chronicity and duration, especially in relations to for example relative risks, where ACE scores were associated to health outcomes. [30–32]. Our aim was to investigate co-occurrence, not to make any claims about the severity of any given ACE itself. In this study, however, we not only assessed individual ACE accumulation scores, we also assessed the ACE accumulation scores for each of the sub-types of ACE separately and provided an overview of the overlap relationships. Our study showed overlap between both the individual ACEs and the three sub-types of ACE, with especially high overlap for neglect and abuse.

### Strengths and limitations

An important strength of this study is that the Young HUNT 3 study was a population study, meaning that the sample was big enough to investigate and compare the individual ACEs as well as ACEs as a whole. Earlier research has mostly focused on individual ACEs [17–19]. In contrast to earlier retrospective research, this study took into account the overlap and accumulation scores of ACEs at the age of 13–19 years [5, 6, 15, 20]. It is intended that prospective research on ACEs in association with other mental health problems will be pursued in this population. The Young HUNT 3 study will then be the largest prospective, population-based, cross-sectional Scandinavian study focusing on ACEs [33]. Because of the used population, the results of this study can be generalized in western world countries as Norway.

An important limitation of the Young HUNT 3 study is that the questionnaires were completed at school, with the inherent difficulties associated with attendance at school which can affect ACE scores, as adolescents who have experienced ACE’s are often more likely to be absent from school. However, 78% of all eligible school children participated in our study.

The Young HUNT 3 study is a population-based study with a broad approach to mental and physical health that has considered questions on a wide scale of topics. ACEs were therefore not defined with the childhood trauma questionnaire but were operationalized afterward. In this context, physical neglect should be mentioned. Neglect was the most challenging ACE to operationalize. Focus with this operationalization was to capture the definition of neglect, the adolescents not being met in their essential needs, as a whole.

However, it should be borne in mind that previous studies using HUNT data have successfully given operational definitions to depression, anxiety and interpersonal problems [34, 35].

## Conclusion

This study shows that the prevalence of ACEs is high with serious interaction, already in adolescence. Especially neglect and abuse show substantial overlap. If ACEs are assessed separately, there is a possibility of obtaining the wrong impression, and feedback to clinical practice about the effects of that ACE could be misleading. This study provides an opportunity to assess those with ACEs, the co-occurrence of ACEs and make correlations with their outcomes later in life.

## Data Availability

The dataset reported in the current article is not publicly available. All data from HUNT are stored at the HUNT research center, with strict rules for access. All data that the researcher uses in their studies are anonymized. Researchers have no access to personal information. It is available on request by qualified scientists. Requests require a concept paper describing the purpose of data access, ethical approval at the applicant’s institution, and provision for secure data access.
